# A technique for taping inferior vena cava caudal to the duodenum: duodenal penetration by IVC filter strut after retroperitoneal lymph node dissection—usefulness of the mesenteric approach

**DOI:** 10.1186/s40792-019-0626-5

**Published:** 2019-04-24

**Authors:** Takayuki Shimizu, Keiichi Kubota, Takashi Suzuki, Takatsugu Matsumoto, Takayuki Shiraki, Yuhki Sakuraoka, Shozo Mori, Yukihiro Iso, Masato Kato, Mitsuru Ishizuka, Taku Aoki

**Affiliations:** Second Department of Surgery, Dokkyo Medical University, 880 Kitakobayashi, Mibu, Tochigi 321-0293 Japan

**Keywords:** Inferior vena cava filter, Retroperitoneal lymph node dissection, Mesenteric approach

## Abstract

**Background:**

Although an inferior vena cava (IVC) filter is used for preventing pulmonary thromboembolism (PTE) in patients with deep vein thrombosis, IVC filter penetration in the duodenum is a rare complication.

**Case presentation:**

A 35-year-old man had previously undergone retroperitoneal lymph node dissection (RPLND) for testicular cancer and IVC filter placement for prevention of PTE. Esophagogastroduodenoscopy (EGD) for his epigastric pain revealed penetration of the IVC filter in the duodenum. The IVC filter was retrieved through cavotomy, and the duodenal penetration was repaired using EGD clipping. Although it was difficult to mobilize the duodenum due to adhesion resulting from RPLND, the use of a mesenteric approach enabled encircling of the IVC caudal to the duodenum. The mesenteric approach is useful and safe for taping the IVC caudal to the duodenum in cases where it is difficult to mobilize the duodenum.

**Conclusion:**

IVC taping using the mesenteric approach allowed safe retrieval of the IVC filter after RPLND without postoperative complications.

## Background

Several reports have demonstrated that inferior vena cava (IVC) filter placement can reduce the incidence of early mortality due to pulmonary thromboembolism (PTE) in patients with deep vein thrombosis (DVT) [1, 2]. IVC filter has been reported to penetrate into the surrounding structures such as the aorta, portal and renal veins, vertebral body, kidney and liver parenchyma, duodenum, large intestine, diaphragm, urinary tract, and retroperitoneum [3]. Although the incidence of perforation of the IVC wall is 0.2% in patients undergoing IVC filter placement [4], the actual incidence of duodenal perforation is unknown. From 1972 to 2017, only 25 cases similar to our case have been reported [3, 5–8]. Herein, we report a very case of duodenal penetration by an IVC filter strut after retroperitoneal lymph node dissection (RPLND).

The patient had a critical surgical problem in that it was difficult to mobilize the duodenum due to a strong adhesion between the IVC and the duodenum as a result of the previous RPLND. Here, we adopted the mesenteric approach to expose and tape the IVC caudal to the duodenum.

## Case presentation

**Fig. 1:**
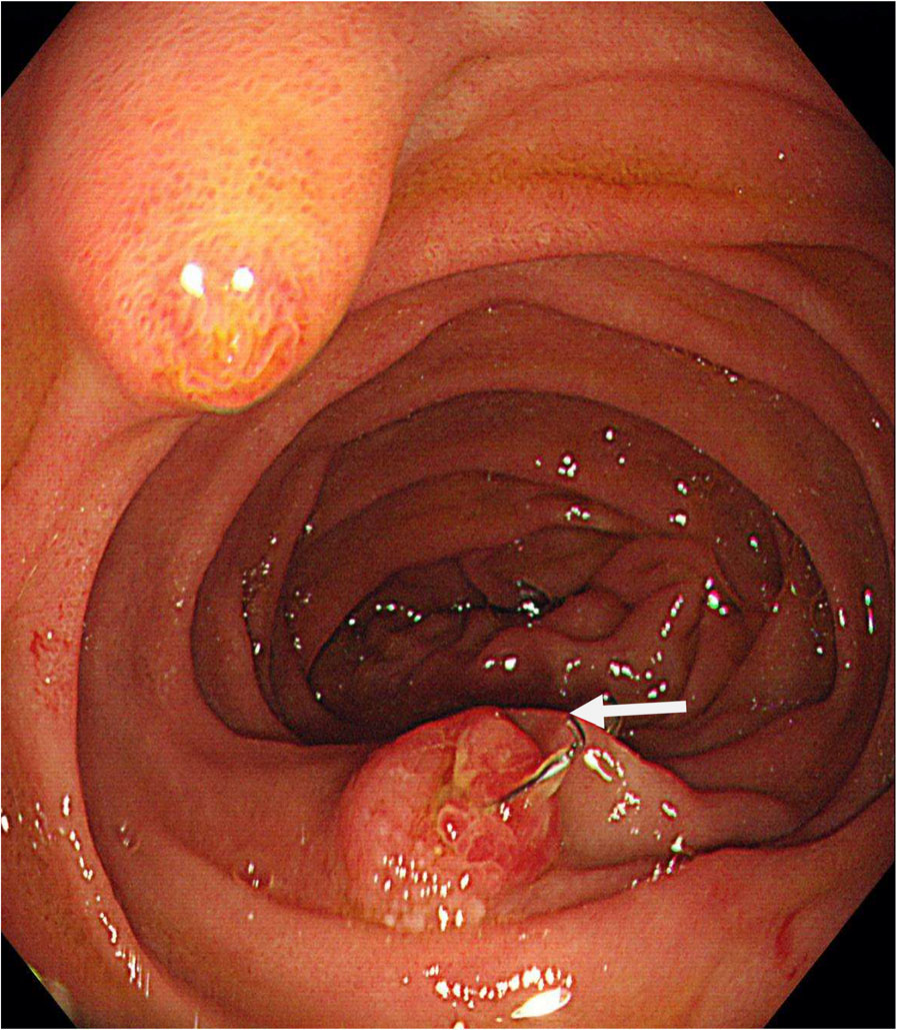
Esophagogastroduodenoscopy (EGD) showed that IVC filter strut penetrated the third portion of the duodenum (arrow)

**Fig. 2:**
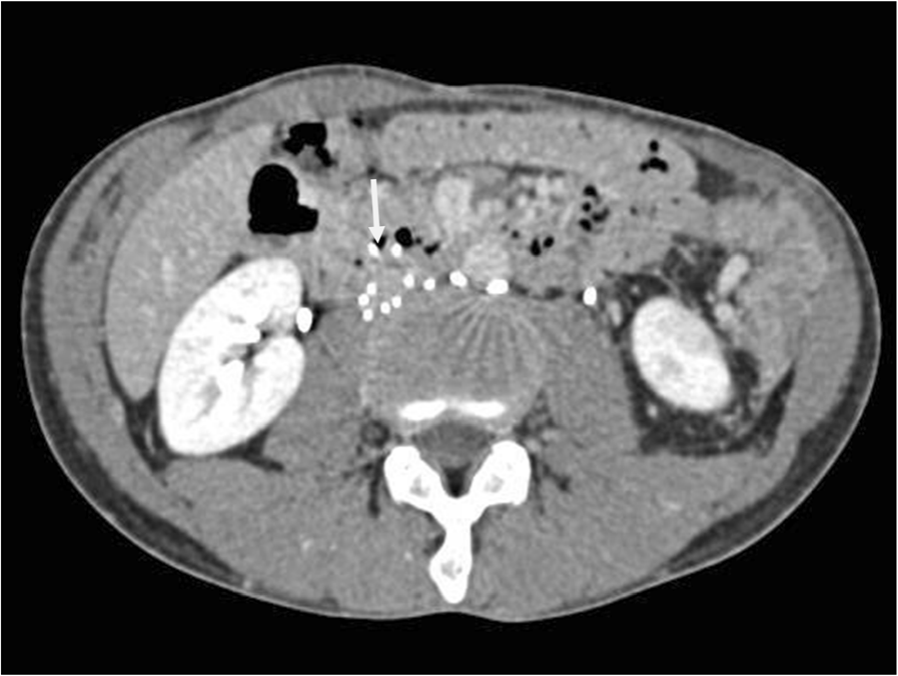
Computed tomography (CT) revealed that IVC filter strut penetrated the third portion of the duodenum (arrow)

A 35-year-old man presented at a local hospital with epigastric pain. Esophagogastroduodenoscopy (EGD) showed that an IVC filter strut had penetrated the third portion of the duodenum (arrow, Fig. 1), and this was confirmed by computed tomography (CT) (arrow, Fig. 2). In order to retrieve the IVC filter, the patient was referred to our department. He had a history of testicular cancer with para-aorta lymph node metastasis. Left renal vein thrombosis developed because of neoadjuvant chemotherapy before RPLND, and anticoagulants were administered before RPLND. Three years previously, he had undergone left orchiectomy, retro-mediastinal lymph node dissection, and RPLND at the previous hospital. The left common iliac vein was intraoperatively damaged during RPLND. Because the previous surgeon was worried about the high incidence of postoperative DVT and PTE, anticoagulant therapy was continued after RPLND. However, because DVT developed in the left common iliac vein after the initial surgery, a retrievable IVC filter (ALN, France) was placed in the IVC caudal to the renal vein to prevent PTE, and the patient had been receiving anticoagulant therapy. Because follow-up CT after IVC filter placement showed that DVT persisted at the left common iliac vein despite anticoagulant therapy, the IVC filter could not be retrieved at the previous hospital.

Enhanced CT also revealed that DVT remained in the left common iliac vein. Because ultrasound examination showed organized DVT, an IVC filter was considered unnecessary. An endovascular approach was considered unfeasible for retrieval because two of the filter struts had penetrated the duodenal wall. An extensive discussion with an internal medicine specialist was performed. Because DVT remained with no remarkable changes in CT images for 3 years and the incidence of PTE caused by DVT in the left common iliac vein would be low, we chose surgical treatment for this patient in order to prevent bleeding at the duodenum.

**Fig. 3:**
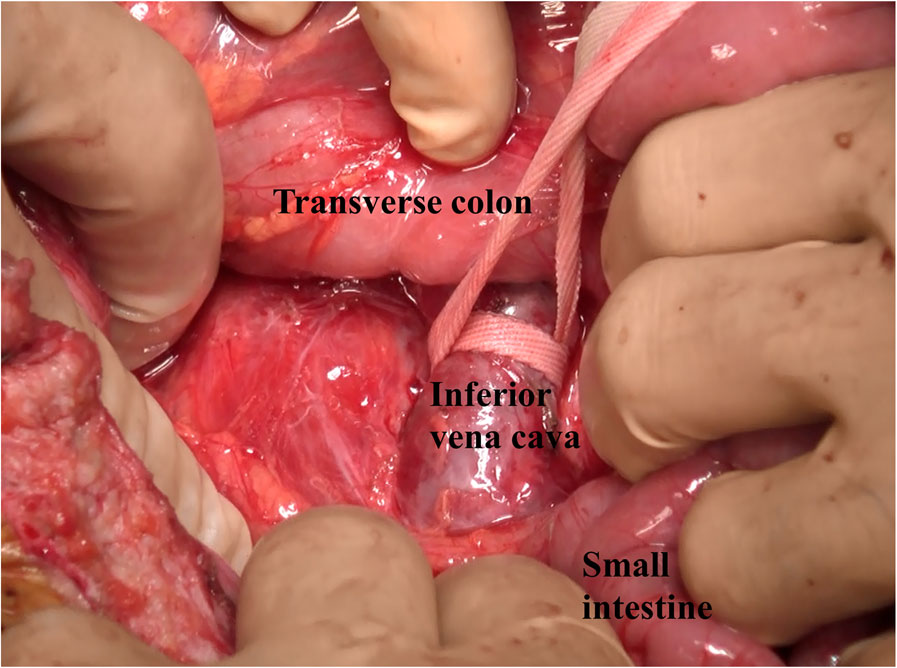
By a mesenteric approach, the IVC could be taped cranial and caudal to the duodenum

**Fig. 4:**
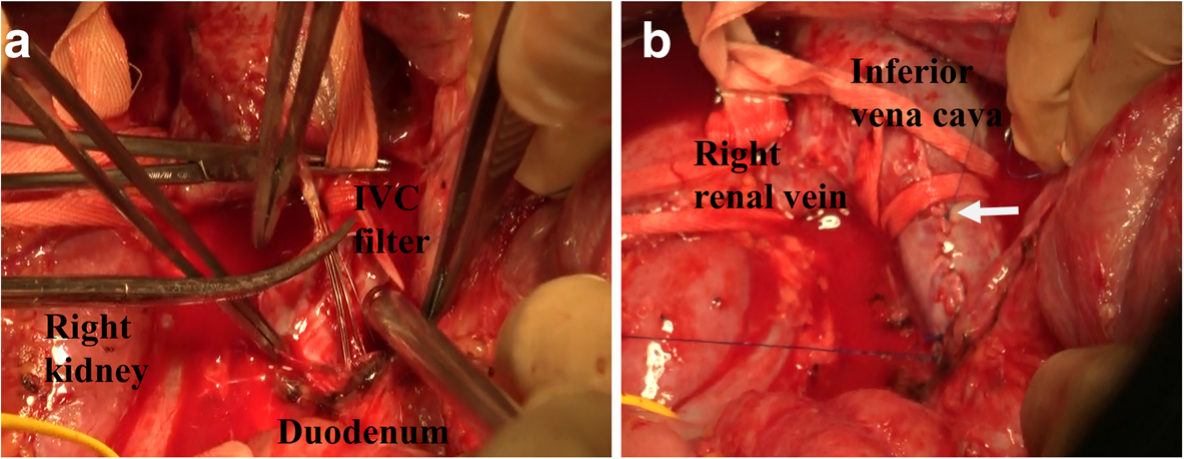
After clamping the IVC cranial and caudal to the duodenum, IVC filter was retrieved from 5 cm vertical incision of IVC cranial to the duodenum (**a**). IVC incision was closed by continuous suture using 5-0 Prolene (arrow, **b**)

**Fig. 5:**
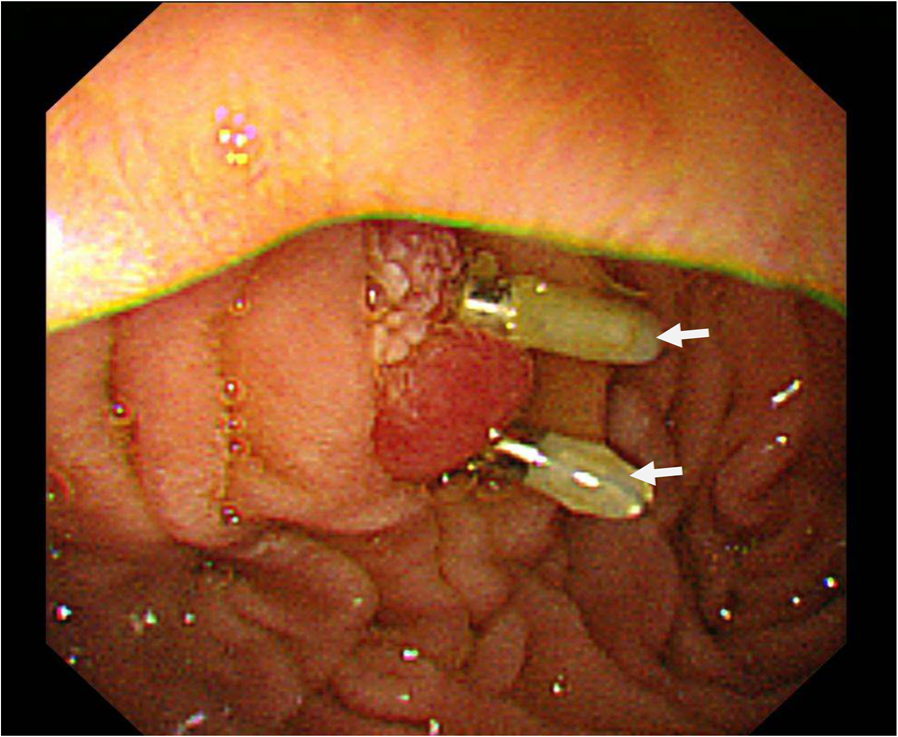
In order to prevent duodenal bleeding or perforation, the penetration site was repaired by EGD clippings (arrows)

**Fig. 6:**
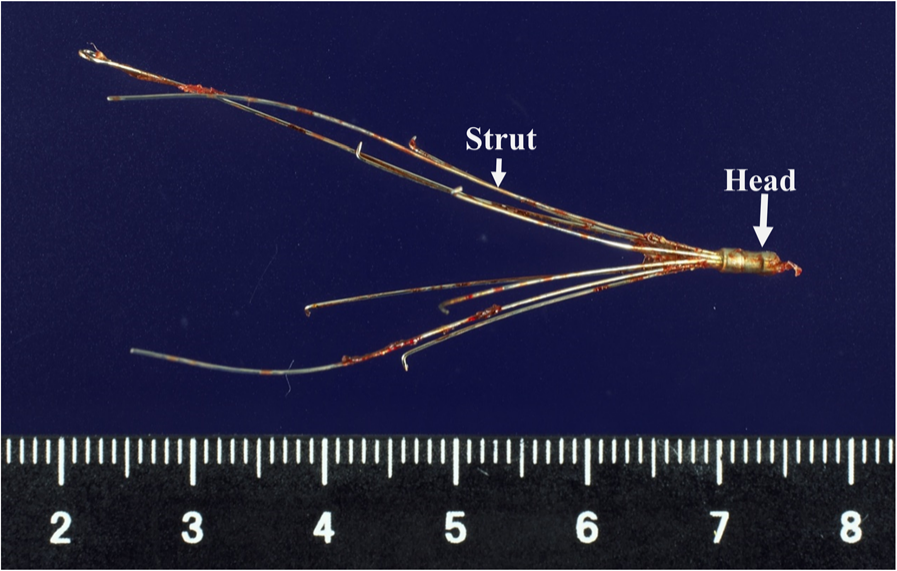
Retrieved IVC filter. The long arrow indicates the head of the IVC filter. The short arrow indicates the strut of the IVC filter

The IVC filter was retrieved through cavotomy, and the duodenal penetration site was repaired using intraoperative EGD clipping. The operation lasted 5 h and 54 min, and the intraoperative bleeding volume was 1172 mL. Because it was not possible to mobilize the duodenum due to adhesions resulting from the previous surgery, the IVC at the sites caudal to the renal vein could not be explored. However, a mesenteric incision caudal to the third portion of the duodenum enabled encircling and taping of the IVC (Fig. 3). After clamping the IVC cranial and caudal to the duodenum, a 5-cm vertical incision was made on the IVC cranial to the duodenum and the IVC filter was retrieved (Fig. 4a). Although the head of the IVC filter had penetrated into the IVC intima, we were able to bluntly peel the filter head from the intima. The IVC incision was closed using a continuous 5-0 Prolene suture (arrow, Fig. 4b). The IVC clamping time was 22 min. Intraoperative EGD revealed no bleeding at the duodenal penetration site (Fig. 5). To prevent duodenal bleeding or perforation, the penetration site was repaired by EGD clipping. Retrieved IVC filter  was presented as (Fig. 6). The patient did not develop any postoperative complications and was discharged on postoperative day 16.

## Discussion

Thirty-two percent of cases of PTE are caused by DVT [9], and the rate of mortality due to symptomatic PTE was 11.9–14.0% [9, 10]. Therefore, prevention of DVT or PTE after surgery is important and can be achieved by anticoagulant therapy and IVC filter placement [11].

It has been reported that preoperative IVC filter placement allowed successful management of RPLND in a case of germ cell tumor-associated IVC thrombosis [12]. However, it has also been reported that an IVC filter placed for preventing PTE 1 month before surgery penetrated the IVC wall [13]. In that case, the IVC was exposed by mobilization of the duodenum under laparotomy, and the incised venous wall was repaired by suturing after retrieval of the filter via an endovascular approach [8]. A recent study has shown that IVC filter placement for 3 months did not reduce the rate of recurrent PTE in patients with DVT and PTE undergoing anticoagulant therapy [14]. The CHEST guideline recommended anticoagulation alone for patients with DVT or PTE [11]. These previous findings suggest that prophylactic IVC placement for DVT or PTE is not recommended in patients who are able to tolerate anticoagulants.

However, in this case, DVT developed in the left common iliac vein after RPLND despite anticoagulant administration for the prevention of DVT and PTE at the previous hospital. Because CHEST guideline recommended the use of an IVC filter in patients with acute DVT or PTE who are treated with anticoagulants [6], the indication of a retrievable IVC filter for this patient was appropriate.

The presented patient had a critical surgical problem in that it was difficult to perform Kocher maneuver due to a strong adhesion between the IVC and the duodenum as a result of the previous surgery, RPLND. Kocher maneuver is useful for the mobilization of the duodenum from the IVC. Although most patients (19/21, 90.4%) reported in a previous systematic review underwent Kocher maneuver [3], the IVC filter had to be retrieved without Kocher maneuver in the present case. Therefore, taping of the IVC caudal to the IVC filter was difficult. Here, we adopted the mesenteric approach to expose and tape the IVC caudal to the duodenum. The mesenteric approach makes it possible to approach the superior mesenteric artery and vein without the Kocher maneuver [15]. In addition, mobilization of the duodenum was unnecessary, thereby avoiding the disruption of the fistula caused by IVC filter penetration and keeping the surgical field clean.

Some reports have indicated that an IVC filter can be retrieved successfully using an endovascular approach in cases where a large hematoma around the duodenum or intra-abdominal adhesion due to a previous surgery precluded surgical retrieval [7, 8]. Although the endovascular approach is less invasive, other reports have documented cases in which endovascular IVC filter retrieval was unsuccessful [5, 6, 16]. In our patient, endovascular IVC filter retrieval might not be possible, because the head of the filter had penetrated the IVC intima and long-term IVC filter placement may have caused filter head penetration into the IVC intima. A systematic review showed that the surgical approach for retrieving IVC filter is associated with a lower rate of complication (1/19, 5.3%), thereby suggesting that a surgical approach is safer [3]. However, an endovascular approach may need to be considered if the surgical approach is not possible.

It is still controversial whether repair of a penetration site in the duodenum is necessary. Although we repaired duodenal penetration by EGD clipping in the present case, duodenal penetration was not repaired in another report with an endovascular IVC retrieval [8]. However, a systematic review revealed that 23.8% of patients (5/21) with duodenal perforation by an IVC filter had symptoms of gastrointestinal bleeding, and 66.7% of patients (14/21) underwent repair of the duodenum [3]. Because the rate of relapse of postoperative gastrointestinal bleeding after retrieval of IVC filter without repair of duodenal penetration remains unclear, it seems prudent to perform repair of duodenal penetration whenever possible.

The systematic review also considered the reason for the increase in the number of cases of duodenal perforation due to IVC filter over the last four decades [3]. The authors considered that prophylactic IVC filter placement for preventing PTE may have been increased over the last two decades and that routine work-up using CT and EGD for abdominal symptoms had become internationally common [3]. Because the frequent chief complaints in patients with duodenal penetration by IVC filter were abdominal pain (11/21) and gastrointestinal bleeding (5/21) [3], EGD was recommended to rule out duodenal perforation due to IVC filter. A retrospective review of CT use concluded that penetration of an IVC filter strut into other organs occurred in 35 of 265 patients (13.2%) [17]. Among them, only 1 patient presented with abdominal pain related to the penetration [17]. However, most patients who had IVC filter penetration into other organs were asymptomatic. Thus, CT and EGD should be performed routinely for patients with IVC filter placement who subsequently develop abdominal symptoms.

In summary, this article has highlighted two important clinical issues: (1) A mesenteric approach is useful and safe for taping the IVC caudal to the duodenum in cases where it is difficult to mobilize the duodenum. (2) If patients can be administered with anticoagulants, the prophylactic IVC filter should be retrieved using an endovascular approach in the early postoperative period. Because prophylactic IVC filter placement for prevention of PTE before RPLND and the routine examination of EGD for abdominal pain will increase the similar reports as our case, our report has a clinical significance in the repair of duodenal perforation caused by IVC filter strut after RPLND.

## Conclusion

Due to IVC taping using mesenteric approach, the retrieving IVC filter after RPLND was safely performed without postoperative complication.

## Abbreviations

CT: Computed tomography
DVT: Deep vein thrombosis
EGD: Esophagogastroduodenoscopy
IVC: Inferior vena cava
PTE: Pulmonary thromboembolism
RPLND: Retroperitoneal lymph node dissection
